# Identification of upregulated exosomal miRNAs between A2780 and A2780/DDP human ovarian cancer cells by high-throughput sequencing

**DOI:** 10.1186/s13048-023-01157-7

**Published:** 2023-05-13

**Authors:** Huihui Wang, Li Liu, Qinying Liu, Jianfeng Zheng, Qiuhong Zheng, Yuwei Chen, Hongmei Xia, Qiaoling Wu, Yang Sun

**Affiliations:** 1 Department of Gynecology, Clinical Oncology School of Fujian Medical University, Fujian Cancer Hospital, No.420 Fuma Road, Jin’an District, Fuzhou, 350014 Fujian Province China; 2 Department of Anesthesiology, Wenzhou Central Hospital, Wenzhou, 325099 Zhejiang Province China; 3 Fujian Provincial Key Laboratory of Tumor Biotherapy, Clinical Oncology School of Fujian Medical University, Fujian Cancer Hospital, Fujian Province 350014 Fuzhou, China; 4 Department of Gynecology, Fujian Cancer Hospital, Fujian Province 350014 Fuzhou, China

**Keywords:** Ovarian cancer, Exosome, microRNA (miRNA), A2780/DDP, Cisplatin resistance

## Abstract

Exosomal miRNAs are known to play important roles in ovarian cancer and chemotherapeutic resistance. However, a systematic evaluation of characteristics of exosomal miRNAs involved in cisplatin resistance in ovarian cancer remains totally unclear. Exosomes (Exo-A2780, Exo-A2780/DDP) were extracted from cisplatin-sensitive cells (A2780) and cisplatin-resistant cells (A2780/DDP). Differential exosomal miRNA expression profiles were found by high-throughput sequencing (HTS). Target genes of the exo-miRNAs were predicted by using two online databases to increase the prediction accuracy. Gene Ontology (GO) and Kyoto Encyclopedia of Genes and Genomes (KEGG) analyses were utilized to find biological relationships with chemoresistance. RT‒qPCR of three exosomal miRNAs was performed, and a protein‒protein interaction (PPI) network was established to identify the hub genes. The GDSC database was used to prove the association between hsa-miR-675-3p expression and the IC50 value. An integrated miRNA–mRNA network was constructed to predict miRNA–mRNA associations. The connection between hsa-miR-675-3p and ovarian cancer was discovered by immune microenvironment analyses. The upregulated exosomal miRNAs could regulate gene targets through signalling pathways such as the Ras, PI3K/Akt, Wnt, and ErbB pathways. GO and KEGG analyses indicated that the target genes were involved in protein binding, transcription regulator activity and DNA binding. The RT‒qPCR results were consistent with the HTS data, and the results of PPI network analysis suggested that FMR1 and CD86 were the hub genes. GDSC database analysis and construction of the integrated miRNA–mRNA network suggested that hsa-miR-675-3p was associated with drug resistance. Immune microenvironment analyses showed that hsa-miR-675-3p was crucial in ovarian cancer. The study suggested that exosomal hsa-miR-675-3p is a potential target for treating ovarian cancer and overcoming cisplatin resistance.

## Background

Ovarian cancer (OC) is the second most common cause of death from gynaecologic cancers in women worldwide [1]. The disease is usually diagnosed late and, even within the same histological subtype, tumours may consist of several subtypes with different biological and molecular characteristics and inconsistencies in the availability and accessibility of treatment. This has resulted in survival rates for OC that have not changed significantly for decades, even in resource-rich countries such as the United States and Canada, and remain at only 47% 5 years after diagnosis (Lheureux2019). Currently, cytoreductive surgery combined with platinum-based chemotherapy is considered the standard treatment option for OC [2]. Since therapeutic strategies are constantly being adjusted, the quality of life of many patients has been improved. However, almost 80% of patients develop cisplatin resistance over the course of treatment, which ultimately leads to death [3]. The recurrence of OC is also associated with the development of drug resistance [4]. Thus, exploring the possible therapeutic targets related to drug resistance mechanisms in OC is a vital undertaking.

MicroRNAs (miRNAs) are small noncoding RNAs (ncRNAs) of approximately 22 nucleotides in length that interact with the 3’ untranslated region (3’-UTR) of a target mRNA, which deregulates the translation or transcription of the target mRNA [5]. The relationships between miRNAs and various cancers have been extensively studied in the past two decades. Based on evidence from these studies of miRNAs, many potential cancer biomarkers have been proposed for diagnostic and prognostic purposes, providing a new perspective for cancer screening [6]. Studies have shown that various miRNAs can influence cisplatin resistance in OC [7]. Zhang et al. found that miR-132 changed the cisplatin sensitivity of OC cells [8]. Jin et al. reported that a miR-149-3p mimic promoted cisplatin resistance in A2780 cells [9]. A recent study demonstrated that miR-30a plays an inhibitory role in DDP resistance via autophagy [10].

Exosomes are spherical vesicles secreted by cells that are 30–140 nm in diameter and are found in a variety of bodily fluids [11, 12]. An abundance of exosomes was found in cisplatin-resistant ovarian cancer cells from female individuals [12]. Recently, miRNAs have been identified in exosomes, whose biogenesis, release, and uptake may involve endosomal sorting complexes (ESCRT complexes) and related proteins required for transport. After release, exosomes are internalized by nearby or distant cells, and the miRNAs contained in them regulate and interfere with tumour immunity and microenvironmental processes, possibly promoting tumour growth, invasion, metastasis, angiogenesis, and drug resistance [13]. For instance, a previous study showed that fibroblast exosomal miRNA, which is associated with lung cancer, could confer cisplatin resistance [14]. More recently, it has been shown that differential expression of exosomal miRNAs plays a crucial role in cisplatin resistance in OC. Hua et al. reported that exosomal miR-98-5p could modulate cisplatin resistance in OC fibroblasts [15]. A previous study showed that exosomal miR-139-5p influenced cisplatin resistance in OC [16].

However, exosomal miRNAs related to cisplatin resistance in OC remain to be further explored, and the mechanism of their influence on cisplatin resistance in OC remains to be studied. The purposes of this study were to find possible biomarkers that influence cisplatin resistance in OC by studying differentially expressed miRNAs in exosomes of the OC cell lines A2780 and A2780/DDP and to investigate the possible mechanisms of their action. We also sought to study whether monitoring the levels of these miRNAs can improve patient outcomes by allowing the prediction of drug susceptibility.

## Methods

### Cell culture

The human OC cell line A2780, a serous cystadenocarcinoma line, was obtained from KeyGEN BioTECH (China), and the human OC cell line A2780/DDP was generated by our laboratory. Both A2780 and A2780/DDP cells were maintained in RPMI-1640 medium (HyClone, USA) supplemented with 10% foetal bovine serum (Gibco, USA) and 1% antibiotic–antimycotic solution (Gibco, USA). Cells were cultured at an optimum temperature of 37 °C and under humidified conditions in the presence of 5% CO_2_.

### Exosome isolation and sample collection

A2780 and A2780/DDP cells were cultured in exosome-free medium, and we collected the cell supernatant 48 h later. First, the cell supernatant was prepared by centrifugation at 3000 × g for 30 min, and then the cells and cell fragments were removed. Next, the Exosome Isolation Kit (from cell culture medium) UR52121 20 T was used. Briefly, 20 ml of cell supernatant was incubated with 5 ml of ExoQuick exosome concentration solution at 4 °C for at least 2 h. Then, centrifugation was performed at 10,000 × g for 60 min to separate and remove the supernatant. The exosomal pellet was resuspended in 200 μl of PBS. The crude exosomes were transferred to an exosome purification filter (EPF column). Centrifugation was performed at 3000 × g for 10 min. The supernatant containing the exosomes was collected and stored at -80℃. Supernatants from the two cell lines were collected for HTS of exosomal miRNAs on the Illumina NextSeq 500 platform (Aksomics Inc., Shanghai).

### Exosome characterization

The morphology of the isolated exosomes was observed by transmission electron microscopy (TEM; Tecnai G2 Spirit 120 kV, USA). In brief, the separated exosomes were mixed with 2% paraformaldehyde and deposited on a copper grid, which was dried for 15 min at room temperature. Furthermore, the samples were stained with 2% uranyl acetate for 10 min and observed by transmission electron microscopy at 120 kV.

### Target gene prediction

MiRDB [17] and TargetScan [18] were utilized to predict the possible target genes of 3 selected upregulated exosomal miRNAs.

### GO functional and KEGG pathway analysis

To investigate the biological functions of upregulated exosomal miRNAs, we performed both GO functional and KEGG pathway enrichment analyses by the clusterProfiler package in R [19].

### Integration of the protein‒protein interaction (PPI) network

The online database STRING and Cytoscape software were utilized to evaluate relevant information on the possible targets of the chosen miRNAs contained in exosomes from various OC cell lines. A combined score of > 0.4 was considered to indicate significance. The hub genes were identified by construction of a PPI network with MCODE.

### RNA extraction and real-time PCR

Total RNA was isolated from ovarian cancer exosomes. One hundred microlitre samples of exosomes were mixed with TRIzol (Tiangen, Beijing). Reverse transcription of miRNAs was performed with the miRNA 1^st^ Strand cDNA Synthesis Kit by stem‒loop reverse transcription (Vazyme, Nanjing). The expression of exosomal miRNAs was normalized to that of miR-425-5p. We utilized miRNA Universal SYBR qPCR Master Mix (Vazyme, Nanjing) to determine the fold changes in the miRNA levels in A2780 exo with respect to those in A2780/DDP exo by the 2 − ΔΔCT method.

### Genomics of Drug Sensitivity in Cancer (GDSC) analysis

Drug IC50 values were predicted by comparing a cell line expression spectrum with gene expression spectrum in TCGA by a ridge regression model (www.cancerrxgene.org/). The R package pRRophetic (version:0.5, https://osf.io/dwzce/? Action = Download) was used to predict the cDDP IC50. The IC50 values were used to divide the miRNAs into two groups (high and low) by expression level. Visual analyses were performed using GraphPad Prism 7.

### Integrated miRNA–mRNA network construction

RNA-Seq data for the TCGA TARGET GTEx cohort was downloaded from UCSC-Xena and UCSC Toil (https://xenabrowser.net/datapages/?dataset=TcgaTargetGtex_rsem_isoform_tpm&host=https%3A%2F%2Ftoil.xenahubs.net&removeHub=http%3A%2F%2F127.0.0.1%3A7222). Data for TCGA-OV samples were obtained from a detailed category named TCGA TARGET GTEx. Then, 427 tumour samples were obtained from the detailed category named “Ovarian Serous Cystadenocarcinoma”. Based on the GENCODE database (httRiskscore://www.gencodegenes.org/), gene annotation information (hg38, GENCODE. V23. annotation. gene. probemap), 60,498 ensembled genes were converted into gene symbols, and low-expression genes were filtered out to retain the genes whose expression value was greater than 0 in more than 1/3 of the samples. After filtering out the low-expression genes, genes with the “protein_coding” annotation were retained as mRNAs. Annotated miRNA information was obtained from the miRbase database (https://www.mirbase.org/).

### Biological function analyses of the immune microenvironment

The stromal and immune scores of samples were estimated based on expression data and the ESTIMATE algorithm indicating the presence of stromal and immune cells. The ESTIMATE scores associated with the two types of cells were used to estimate tumour purity. The R package GSVA (version: 1.36.2, http://bioconductor.org/packages/release/bioc/html/GSVA.html) based on the ssGSEA (single-sample enrichment analysis) algorithm was used, and the enrichment scores of immune cells were also calculated to indicate the relative abundances of each type of TME-infiltrated cell. The gene set identified 28 types of TME-infiltrated immune cells. CIBERSORT (httRiskscore://cibersort.stanford.edu/index.php) allows the proportions of 22 kinds of immune cells to be computed. CIBERSORT is a tool used for deconvolution of the expression matrix of immune cell subtypes based on linear support vector regression. MCPcounter is an absolute counting method implemented in the R package MCPcounter (httRiskscore://github.com/ebecht/MCPcounter) that is based on the complete mRNA expression matrix. The relative abundances of 9 types of infiltrating immune cells in each sample were estimated. The xCell tool integrates the ssGSEA method for cell type enrichment analysis based on gene expression data of 64 immune and stromal cell types. The online web tool xCell (httRiskscore://xcell.ucsf.edu/) was used to enter the complete mRNA expression matrix and estimate the relative abundances of immune cells and stromal cells in the various samples. On the basis of miRNA expression groups, the differences in the proportions of various immune cells were compared, and a box plot was drawn.

## Results

### Identification of exosomal miRNAs

**Fig. 1:**
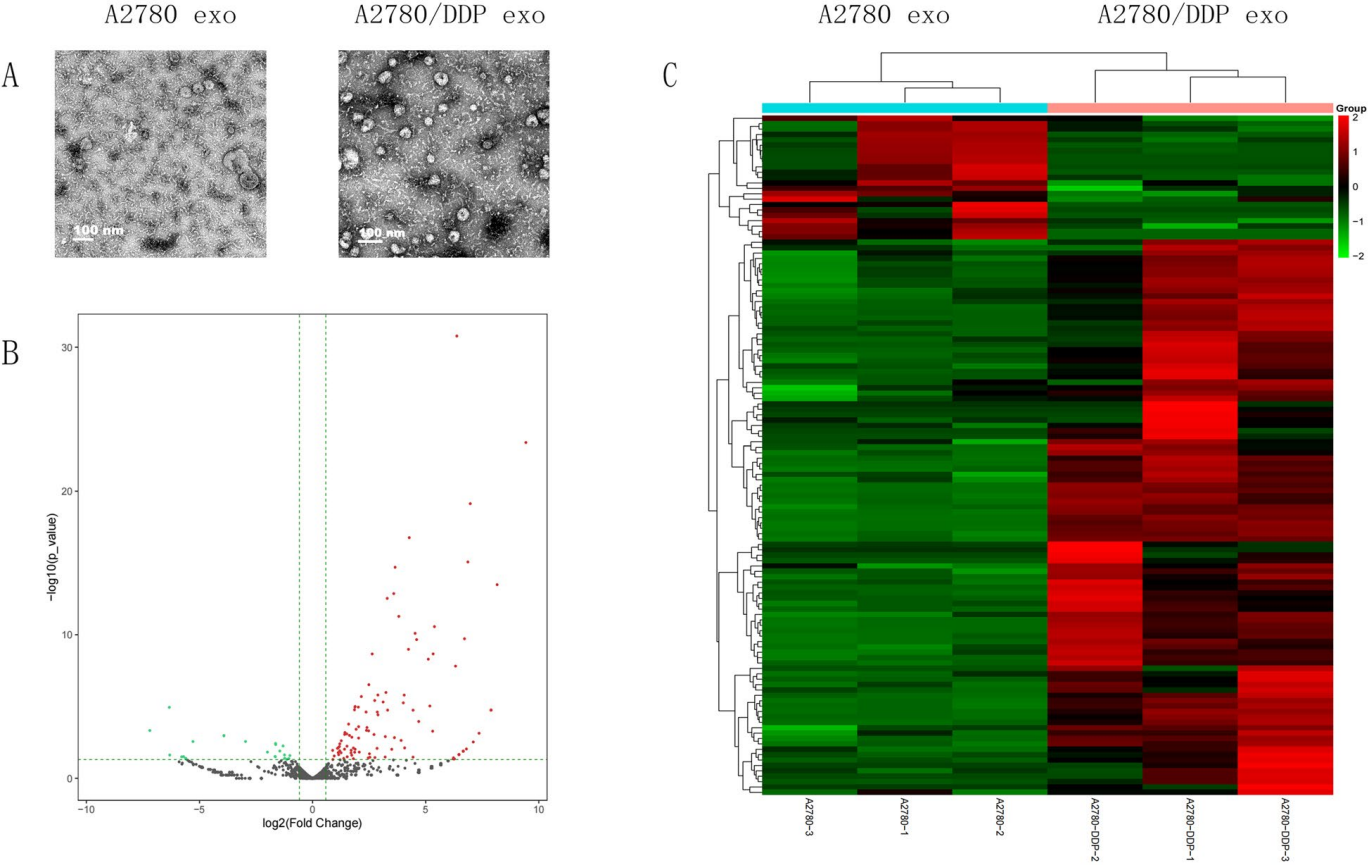
Characterization of exosomes and their DE-miRNAs (**A**) A2780 exo and A2780/DDP exo were identified through TEM to have diameters of 30–130 nm (Scale bar, 100 nm). **B** Volcano plot of differentially expressed miRNAs. Red indicates the upregulated miRNAs between A2780 exo and A2780/DDP exo, green indicates the downregulated miRNAs, and black indicates that no difference in expression. **C** Hierarchical heatmap showing differential miRNA expression identified by high-throughput sequencing (HTS). Red and green indicate the different expression levels in the colour scale

A2780 exo and A2780/DDP exo were validated by TEM to have diameters ranging from 30 to 130 nm (Fig. 1A). HTS was performed on OC cell-derived exosomal miRNAs isolated from exosomes derived from two OC cell lines. A total of 103 miRNAs were upregulated and 23 miRNAs were downregulated in A2780/DDP exo compared with A2780 exo. The DE-miRNA identification thresholds were a fold change of at least 2 and *P* < 0.05. The top ten upregulated exosomal miRNAs are listed in Table 1. Then, we selected three mature miRNAs related to chemoresistance. The targets of 1431 exosomal miRNAs were predicted by miRDB and TargetScan.

**Table 1:** Top 10 upregulated miRNAs between A2780 exo and A2780/DDP exo

miRNA	Log_2_FC	P	Fold Change	Expression
hsa-miR-675-3p	9.429868577	4.32438E-24	689.7207581	Up
hsa-miR-7704	8.158181287	3.24576E-14	285.6651606	Up
hsa-miR-200b-5p	7.893775274	1.76314E-05	237.8280836	Up
hsa-miR-675-5p	7.883877891	1.79375E-05	236.2020852	Up
hsa-miR-1266-5p	7.360302372	0.00072884	164.3129493	Up
hsa-miR-10399-5p	7.103450601	0.002939239	137.5155163	Up
hsa-miR-552-5p	6.969611185	7.39091E-20	125.3320158	Up
hsa-miR-194-3p	6.864683335	8.58681E-16–16	116.5401558	Up
hsa-miR-371a-5p	6.800425889	0.009038524	111.4633717	Up
hsa-miR-429	6.716542787	1.89701E-10–10	105.1673295	Up

### Ten differentially upregulated exosomal miRNAs and cisplatin resistance

**Fig. 2:**
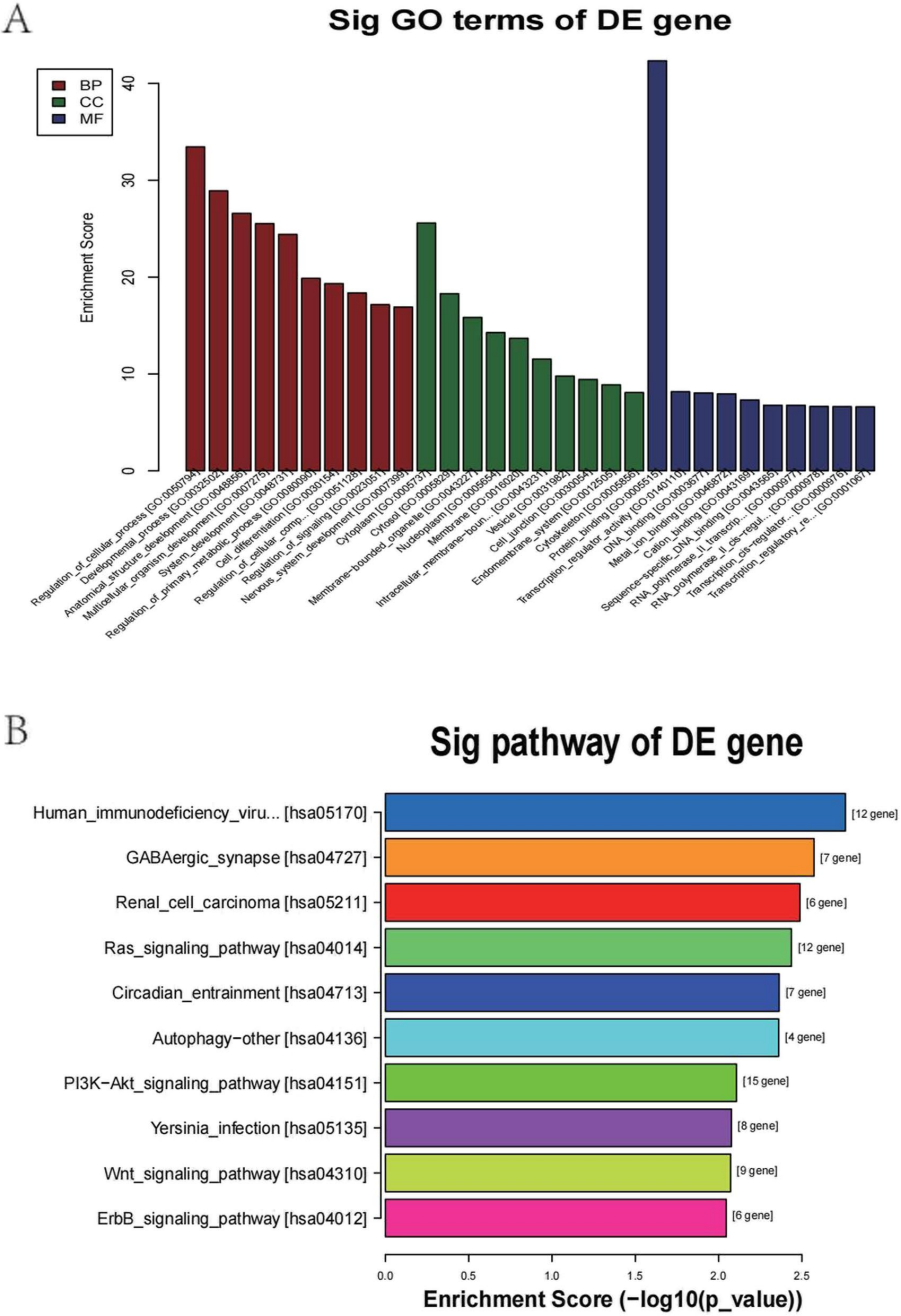
GO and KEGG analyses of the top 10 miRNAs with differential expression. Only the enriched pathways with *P* < 0.05 are shown

**Fig. 3:**
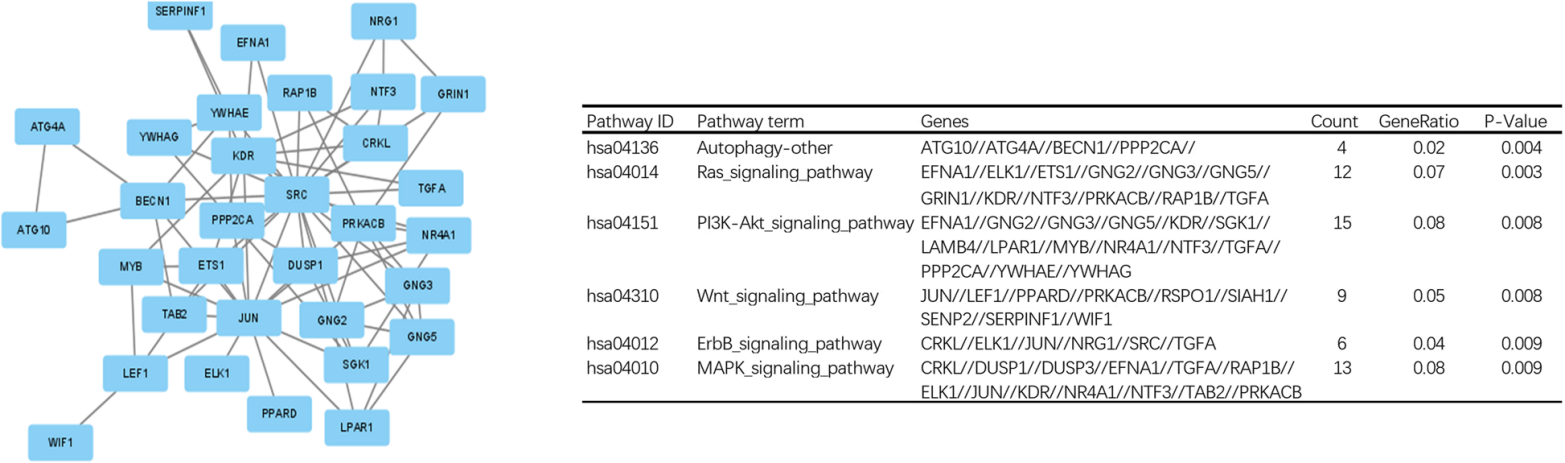
Protein‒protein interaction network analysis showing related modules and enriched pathways

To explore the functions of the targets of the 10 predicted exosomal miRNAs, GO biological process (BP), cell component (CC) and molecular function (MF) analyses were performed (Fig. 2A). Regulation of cellular process, developmental process, anatomical structure development and so on were identified in the BP category. Cytoplasm, cytosol, membrane-bounded organelle and so on were identified in the CC category. Protein binding, transcription regular activity, DNA binding and so on were identified in the MF category. In investigating the associated pathways of the predicted exosomal miRNA targets, KEGG pathway analysis showed that 10 main pathways were involved. The pathways named Ras signalling pathways, autophagy–other, PI3K-Akt signaling pathway, Wnt signaling pathways and ErbB-signaling pathway were identified by KEGG analysis (Fig. 2B). Most of these pathways were related to cisplatin resistance. The OC-related pathways and their modules were determined by constructing the PPI network in STRING. The PPI network contained 30 genes, as shown in Fig. 3.

### Three mature exosomal miRNAs in the A2780/DDP cell exosomal supernatant

**Fig. 4:**
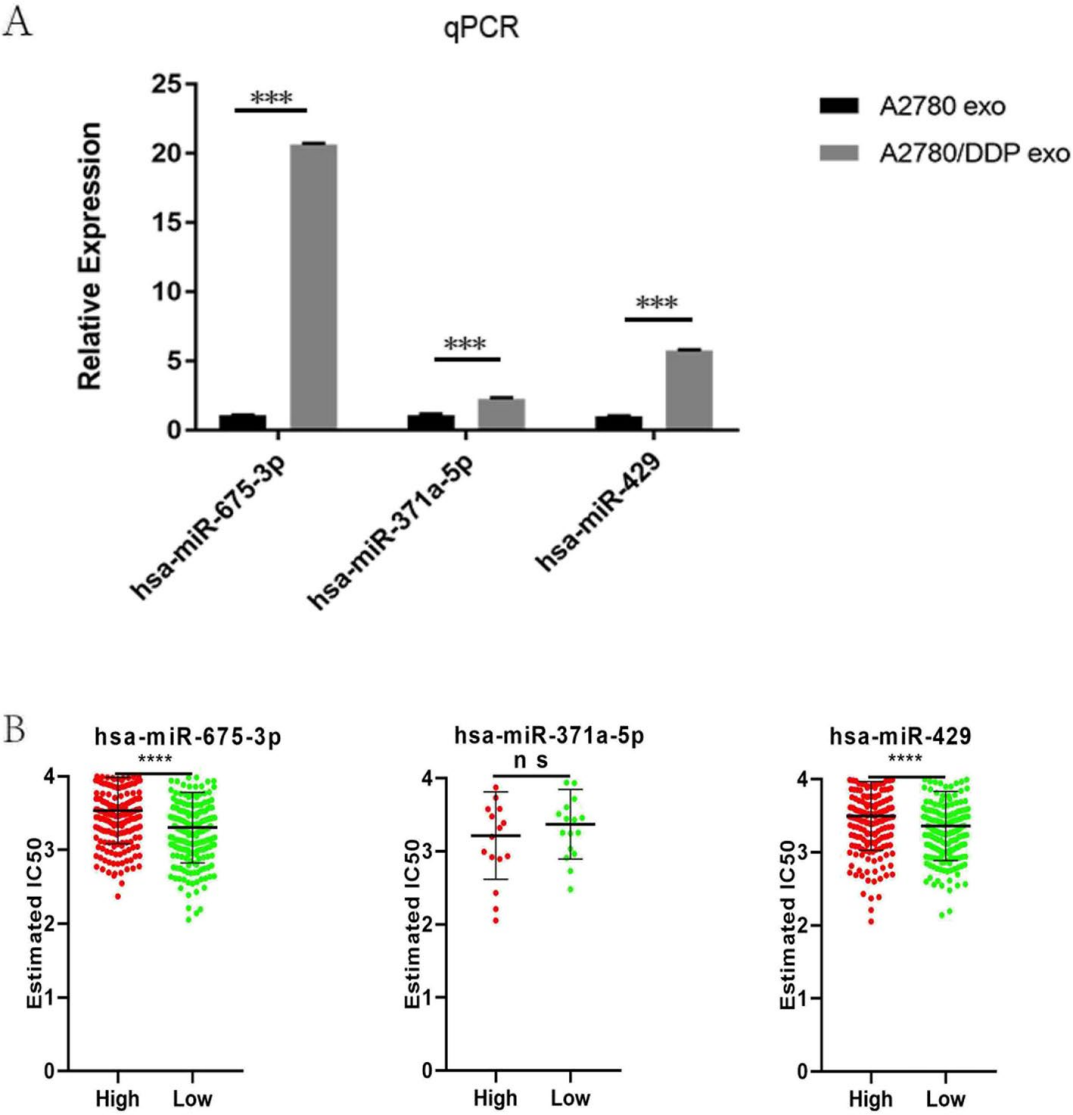
Expression levels of three miRNAs and their relationship with IC50 values. **A** Expression of hsa-miR-675-3p, hsa-miR-371a-5p and hsa-miR-429 in exosomes derived from the ovarian cancer cell lines A2780 and A2780/DDP. Data are expressed as the mean ± standard deviation of three independent experiments (*P *< 0.05). **B** Differential IC50 levels in the high and low hsa-miR-675-3p, hsa-miR-371a-5p and hsa-miR-429 expression groups in ovarian cancer in the GDSC database

Three mature exosomal miRNAs were selected and identified by RT‒qPCR (Fig. 4). Hsa-miR-675-3p, hsa-miR-371a-5p and hsa-miR-429 were upregulated in A2780/DDP exo compared to A2780 exo (*P* < 0.05), consistent with the HTS data. In summary, hsa-miR-675-3p had the highest expression level among the 3 miRNAs in A2780/DDP exo, and it may thus play an important role in cisplatin resistance.

### Exosomal hsa-miR-675-3p and cisplatin resistance

To investigate the roles of miRNAs in ovarian cancer, the Genomics of Drug Sensitivity in Cancer (GDSC) database was used to predict the associations between miRNA expression levels and IC50 values. Figure 4 shows that high expression of hsa-miR-675-3p and hsa-miR-429 was consistent with a high IC50 value.

### The cisplatin resistance genes and mRNA‒miRNA network of hsa-miR-675-3p

**Fig. 5:**
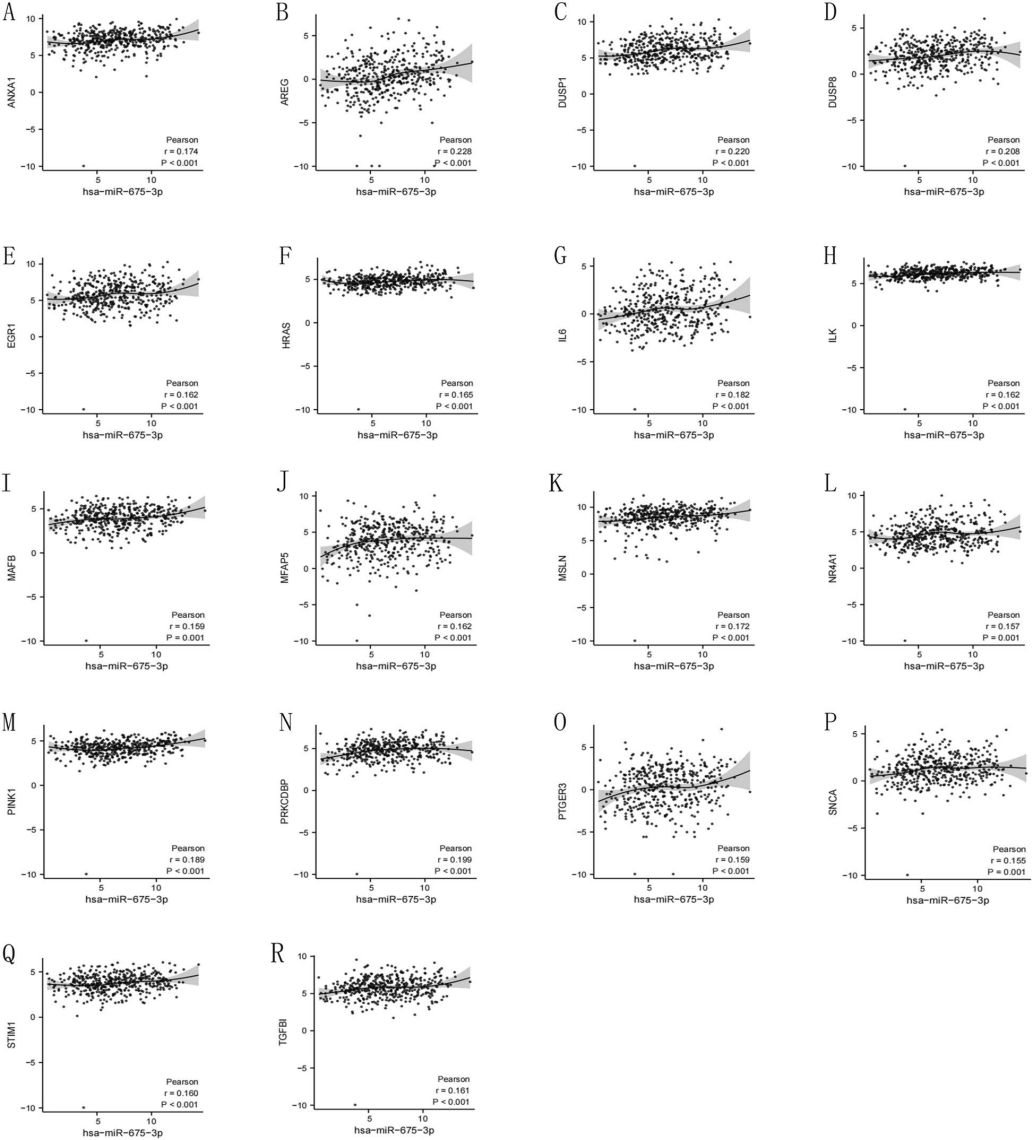
The links between hsa-miR-675-3p and its associated genes in ovarian cancer cisplatin resistance

**Fig. 6:**
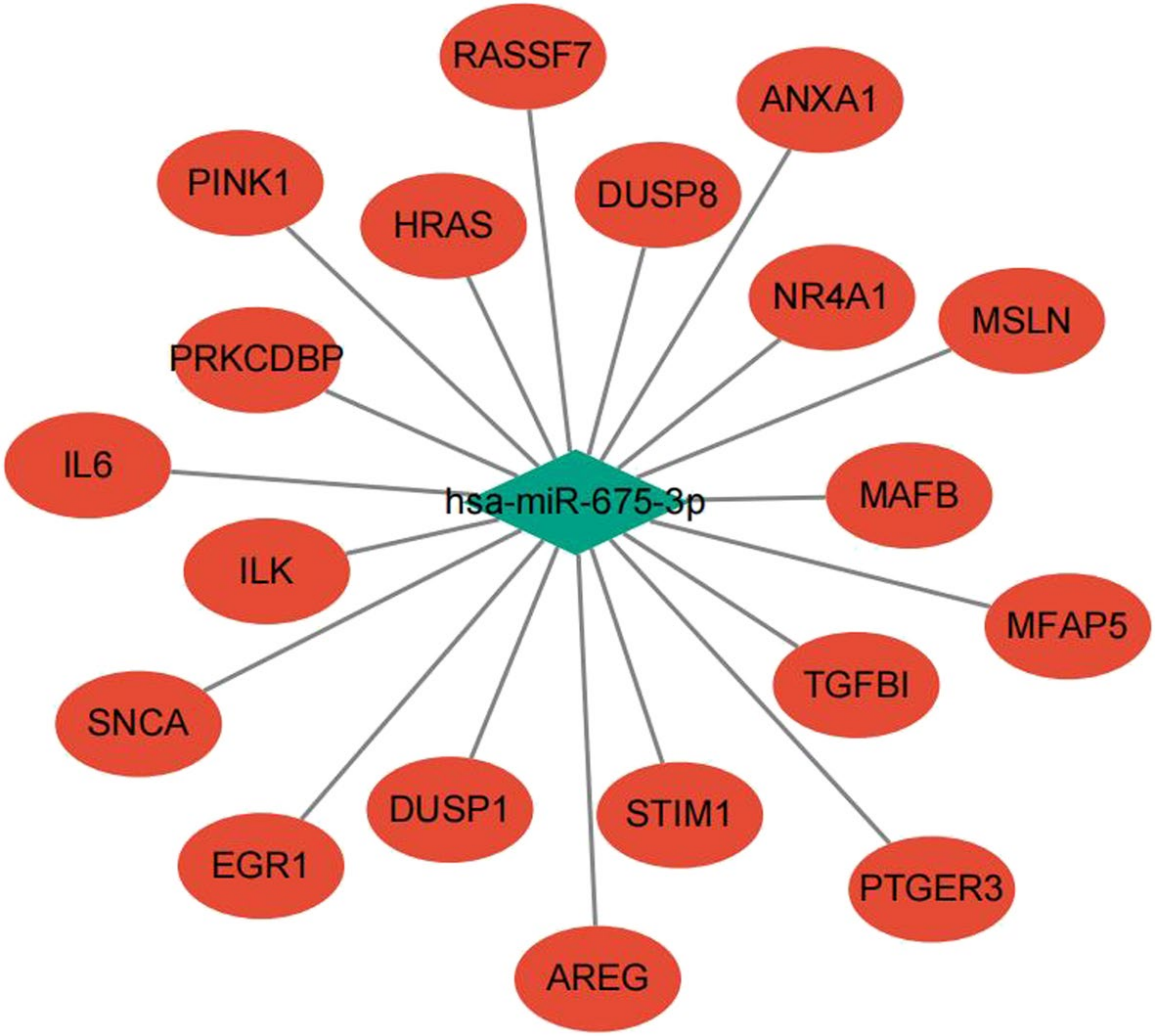
The integrated miRNA–mRNA network of ovarian cancer cisplatin resistance

To explore the associations of miR-675-3p with its hub genes, PPI network analysis was performed to identify the genes listed in Table 2 (FMR, CD86, DDX3X, EIF4E, CHEK1, RAB5A, PIWIL1, RASGRF1, SCN9A, SYNPR). The statistical data on cisplatin resistance protein expression used to build the mRNA‒miRNA network was obtained from the TCGA database. The associated genes (AREG, DUSP1, DUSP8, EGR1, HRAS, IL6, ILK, MAFB, MFAP5, MSLN, NR4A1, PINK1, PRKCDBP, PTGER3, SNCA, STIM1 and TGFBI) were selected, and their relationships with miR-675-3p are vividly shown in Fig. 5. The miRNA–mRNA network was constructed to reveal the relationship between miR-675-3p and cisplatin resistance in ovarian cancer (Fig. 6).

**Table 2:** Hub genes of hsa-miR-675-3p involved in PPIs

Name	Score
FMR1	8
CD86	8
DDX3X	7
EIF4E	7
CHEK1	7
RAB5A	6
PIWIL1	6
RASGRF1	6
SCN9A	5
SYNPR	5

### Associated immune microenvironment of hsa-miR-675-3p

**Fig. 7:**
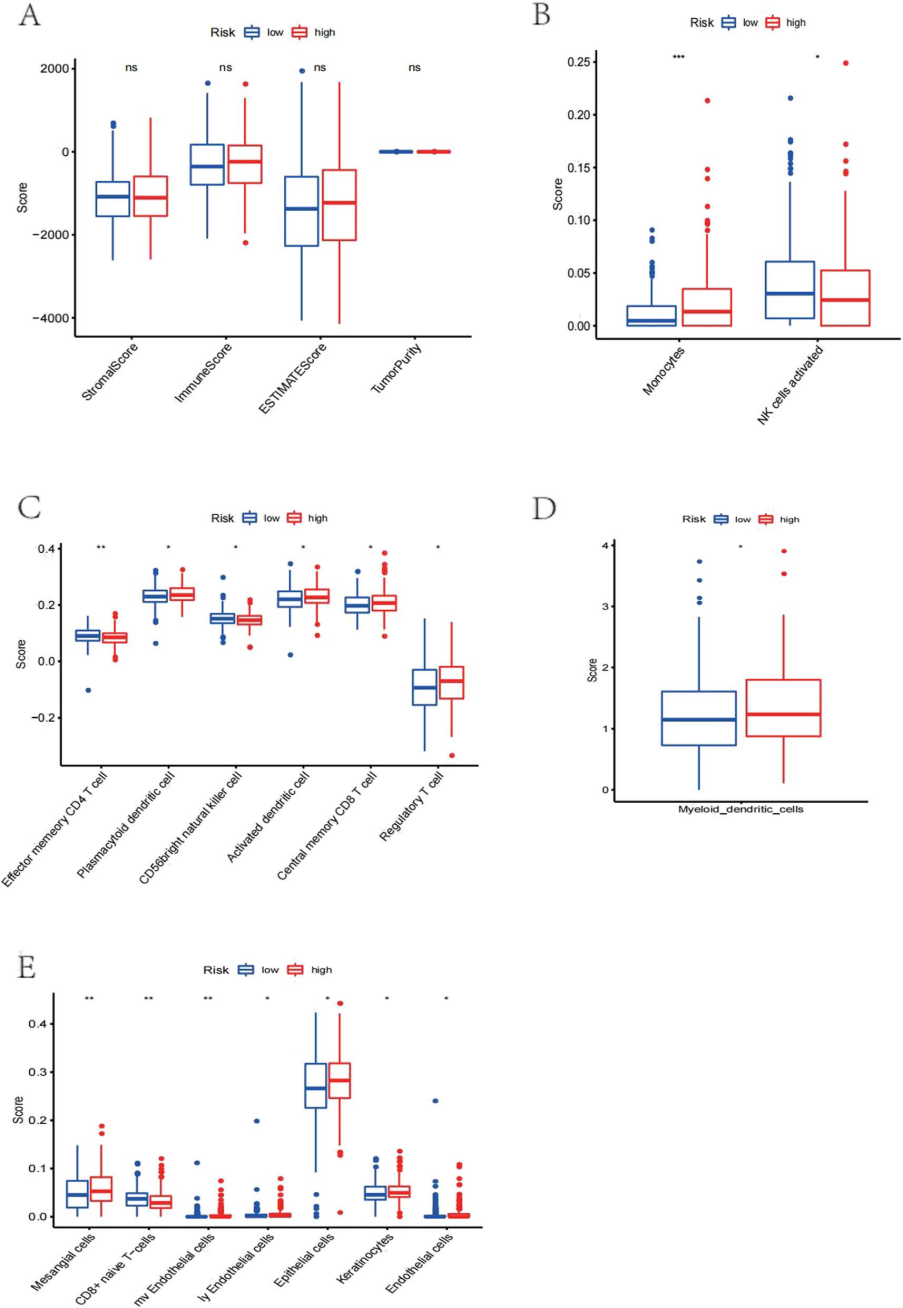
Assessment of the immune microenvironment and its relationship to hsa-miR-675-3p

With five algorithms, two groups were comprehensively evaluated. The results revealed differences in the stromal score, immune score and ESTIMATE score between the groups stratified by hsa-miR-675-3p expression (Fig. 7A). In addition, high abundances of monocytes and activated NK cells were identified in the high hsa-miR-675-3p expression group (Fig. 7B). Among these immune cells, plasmacytoid dendritic cells, CD56 bright natural killer cells and central memory CD8 T cells were three most abundant cell types in the high-risk group (Fig. 7C and D). Finally, upregulated hsa-miR-675-3p showed the highest score in epithelial cells (Fig. 7E).

## Discussion

As one of the most common female reproductive system carcinomas, ovarian cancer is often found late but has a high relapse rate [20]. Cisplatin resistance is the major barrier to the treatment of ovarian cancer [21]. For the success of chemotherapy, it is critical to confirm the functions of exosomal miRNAs [22]. A previous study demonstrated that docetaxel-resistant cells (MCF-7/Doc) and parental MCF-7 cells (MCF/S) had differences in crucial pathways and biological functions in breast cancer chemoresistance [23]. DE-miRNAs in ovarian cancer exosomes were found by expression profiling, and relevant bioinformatic analysis was performed to explore their biological functions. To the best of our knowledge, this is the first bioinformatic analysis of upregulated miRNAs in cisplatin-resistant cell-derived exosomes (A2780/DDP exo) and parental cisplatin-sensitive cell-derived exosomes (A2780 exo). The potential targets of cisplatin resistance in ovarian cancer cell-derived exosomes are of great importance to explore.

In the present study, exosomal miRNA expression profiles of A2780/DDP and A2780 cells were analysed by high-throughput sequencing (HTS). A total of 103 upregulated miRNAs and 23 downregulated miRNAs were identified in A2780/DDP exo compared to A2780 exo. In total, 2764 exosomal miRNA targets in A2780/DDP cells were found by using miRDB and TargetScan. The interactions among these target genes were investigated by GO and KEGG analyses. The identified BP terms were related to the pathways named regulation of cellular process, developmental process, anatomical structure development and so on. The identified CC terms were associated with cytoplasm, cytosol membrane-bounded organelle and so on. The identified MF terms were related to cytoplasm, cytosol, membrane-bounded organelle and so on.

MiR-675-3p was the most upregulated miRNA in A2780/DDP exo, as determined by RT‒qPCR.

A previous study showed that miR-675-3p could enhance the migration and invasion capacities of oesophageal cancer cells [24]. A recent study showed that exosomal miR-675-3p accelerated cisplatin resistance in gastric cancer in vivo [25]. However, the role of hsa-miR-675-3p in A2780/DDP exo and A2780 exo has not been clearly investigated. Moreover, exosomal miR-429 confers chemoresistance on the epithelial ovarian cancer cell lines SKOV3 and A2780 [26]. Therefore, it is of great importance to explore the selected exosomal miRNAs and their relationships with cisplatin resistance in ovarian cancer cell lines.

Differentially upregulated exosomal miRNAs were shown in our study, especially exosomal hsa-miR-675-3p in the OC cell line A2780/DDP exo. Additionally, the hub genes FMR1 [27] and CD86 [28] are closely connected to hsa-miR-675-3p. The mRNA‒miRNA network was constructed to explore cisplatin resistance genes. ANXA1 [29], AREG [29], DUSP1 [30], DUSP8 [31], EGR1 [32], HRAS [33], IL6 [34], ILK [31], MAFB [35], MFAP5 [36], MSLN [37], NR4A1 [38], PINK1 [39], PRKCDBP [40], PTGER3 [41], SNCA [42], STIM1 [43] and TGFBI [44] have all been proven to be associated with cisplatin resistance. Immune environment analyses proved the connection of miR-675-3p to immune cells, and our data also showed that high expression of miR-675-3p might be related to decreased sensitivity to cisplatin. These results indicated that hsa-miR-675-3p may be the link between ovarian cancer and cisplatin resistance. More experiments should be performed to explore the underlying mechanism.

The research we performed still has limitations. First, we used only two cell lines in our HTS analysis. Therefore, follow-up requires more time and samples. In addition, the mechanisms of DE-miRNAs in OC exosomes needs to be further explored. Consequently, more efforts should be made to overcome these difficulties in the laboratory.

## Conclusions

In brief, we provided a comprehensive analysis of upregulated exosomal miRNAs between cisplatin-resistant ovarian cancer cells (A2780/DDP) and parental cisplatin-sensitive ovarian cancer cells (A2780). GO, KEGG, PPI network, RT‒qPCR, and GDSC analyses, as well as cisplatin resistance gene predictions and immune analyses, were performed after HTS. These studies of exosomal miRNAs revealed differences in cisplatin resistance in ovarian cancer. Laboratory research based on hsa-miR-675-3p in ovarian cancer cell-derived exosomes is needed to explore its specific mechanism in cisplatin resistance.

## Data Availability

The raw HTS data have been submitted to the NCBI SRA. The accession Numbers were:

## Abbreviations

miRNA: MicroRNA
DE-miRNAs: Differentially Expressed miRNAs
GO: Gene Ontology
KEGG: Kyoto Encyclopedia of Genes and Genomes
OC: Ovarian cancer
TEM: Transmission Electron Microscopy
IC50: Half-maximal inhibitory concentration
